# An Analytical Protocol for the Differentiation and the Potentiometric Determination of Fluorine-Containing Fractions in Bovine Milk

**DOI:** 10.3390/molecules28031349

**Published:** 2023-01-31

**Authors:** Nadia Spano, Sara Bortolu, Margherita Addis, Ilaria Langasco, Andrea Mara, Maria I. Pilo, Gavino Sanna, Pietro P. Urgeghe

**Affiliations:** 1 Dipartimento di Scienze Chimiche, Fisiche, Matematiche e Naturali, Università degli Studi di Sassari, Via Vienna, 2, I-07100 Sassari, Italy; 2 Istituto per la Bioeconomia, Consiglio Nazionale delle Ricerche, Traversa La Crucca, 3, I-07100 Sassari, Italy; 3 Agris Sardegna, Loc. Bonassai Km.18,600, I-07040 Olmedo, Italy; 4 Dipartimento di Agraria, Università degli Studi di Sassari, Viale Italia 39A, I-07100 Sassari, Italy

**Keywords:** fluorine, fluoride, milk, potentiometry, ion specific electrode, caries

## Abstract

Free fluoride ions are effective in combating caries in children, and their supplementation in milk has been widely used worldwide for this purpose. Furthermore, it is known that ionic fluoride added to milk is distributed among its components, but little is known about their quantitative relationships. This is likely due to the absence of an analytical protocol aimed at differentiating and quantifying the most important forms of fluorine present in milk. For the first time, a comprehensive protocol made up of six potentiometric methods devoted to quantifying the most important fractions of fluorine in milk (i.e., the free inorganic fluoride, the inorganic bonded fluorine, the caseins-bonded fluorine, the whey-bonded fluorine, the lipid-bonded fluorine, and the total fluorine) has been developed and tested on real samples. Four of the six methods of the procedure are original, and all have been validated in terms of limit of detection and quantification, precision, and trueness. The data obtained show that 9% of all fluorine was in ionic form, while 66.3% of total fluorine was bound to proteins and lipids, therefore unavailable for human absorption. Beyond applications in dental research, this protocol could be extended also to other foods, or used in environmental monitoring.

## 1. Introduction

Fluorine is the 13th most common element in the Earth’s crust, with a mean concentration of 600–900 mg kg^−1^ [1]. Molecular fluorine is rarely present in nature [2] whereas fluoride, its reduced form, is the nearly ubiquitous chemical form in water, soil, plants, and animals. Although the ‘essentiality’ of fluoride for the biosphere has been debated for many decades [3], its role for humans has only been clearly established in recent years. Based on the European Food Safety Authority (EFSA) [4], fluoride is not an essential species for humans since no health consequences of its deficiency have been observed. Conversely, excess fluoride has been found to cause dental fluorosis [5,6] or skeletal fluorosis [7], gastric and kidney disturbances [8], or even death [9,10]. On the other hand, a moderate intake of fluoride is helpful in combating the development of caries in children [11,12]. Free fluoride is the most active species in caries prophylaxis. It is incorporated in the hydroxyapatite of developing tooth enamel and dentin, forming fluorohydroxyapatite, which is more acid-resistant than hydroxyapatite. In addition, fluoride has an anticaries effect on erupting teeth by contact with enamel during ingestion, excretion in the saliva, and absorption in biofilms on teeth [13]. Even for this reason, the European Union has set the recommended daily value for fluoride intake at 3.5 mg [14].

Although ionic fluoride is the only chemical form of fluorine present in non-polluted water, this element is present in biological samples in two main forms: inorganic fluorides and “organic” (or “covalent”) fluorine [15]. The inorganic fraction is represented by free ionic fluoride (FIF), i.e., the only chemical form which is detected by the fluoride ion selective electrode (FISE), and by inorganic bonded fluoride (IBF). The latter form is represented by (i) H-bonded compounds (i.e., HF), (ii) metal-bonded compounds (e.g., fluoride salts or their metal complexes), (iii) fluoride “adsorbed” by mineral/organic sediments, and (iv) inorganic fluoride incorporated into biological tissues such as teeth or bones [15]. In the past, IBF has been conveniently determined by using microdiffusion procedures [16,17,18,19,20]; at very low pH values, all inorganic forms of fluoride were converted to HF, which diffused into the hexamethyldisiloxane (HMDS) medium and quantitatively recovered in an alkaline solution. For this reason, the sum of the inorganic fluorides (TIF) has also been called “diffusible” fluoride.

Beyond inorganic fluorides, at least one further form of fluorine, first revealed by Taves in 1968 [21], is present in biological samples: the so-called “organic” fluorine, as previously defined by Venkateswarlu [15]. Although the nature of the bond (or interaction) between fluorine and the organic matrix has not been well established yet, the “organic” fluorine is characterized by substantial inertia towards the adsorption/exchange phenomena involving that element. Consequently, organic fluorine does not readily release fluorides, such as HF or calcium phosphate, under acidic conditions or in exchange processes with the strong adsorbent material, respectively [21].

Among the possible fluorine sources, diet is the primary one of intake for humans. Almost all foods contain this element. Unpolluted water may contain up to a few mg dm^−3^ of fluoride [7], whereas the concentration of this species in the edible part of some plants, such as grapes, spinach, elderberry, and-mainly- tomato and tea, may exceed the level of the g kg^−1^ as a function of the geochemical threshold of the soil where they are grown [8]. Since the level of fluoride in drinking water may be insufficient to achieve the outcomes in childhood caries prevention, strategies for fluoride supplementation have been adopted in previous decades [12]. Fluorination of water [22] and dietary fluoride supplementation in salt [23] or milk [24] have been widely used to carry out this activity.

Milk is the primary food during the early years of human life. The fluoride content of milk could play a role in improving the mineralization of teeth in children and preventing dental caries. Consequently, the consumption of fluoridated milk has been successfully promoted in many countries [24,25,26,27,28,29,30,31,32]. As a result, several authors have developed analytical methods aimed at assessing the fluoride levels in milk-based matrices. Among others, cow milk [33,34,35,36,37,38,39,40,41,42,43,44,45,46,47,48,49,50,51,52], breast milk [47,53,54,55,56,57,58], and infant milk [59,60], or formulas, [47,55,61,62] were primarily studied using fluorescence [18], molecular absorption [38,49,52] or atomic [50] spectrometry, gas-chromatography [39,42], ion chromatography [48] and, among all, potentiometric methods with ion-specific electrodes (ISE) [15,18,20,33,34,35,36,37,40,41,43,44,45,46,47,51,53,54,55,56,57,58,59,60,61,62]. In this technique, the sensing element is a crystal of LaF_3_ doped with EuF_2_. The lattice vacancies in this way created an increase in the mobility of fluoride ions that jump between them. Since the crystal membrane is permeable only to fluoride ions, the potential of the electrode depends only on their activity [63]. The sensor is almost specific, being that the only interfering ion is OH^−^. To overcome this potential interference, and to avoid the protonation of the analyte, FISE measurements must be performed at pH between 5 and 6.

The methods commonly reported in the literature are aimed at the determination of free or diffusible fluoride, and total fluorine in milk samples [33,34,35,36,37,41,44,46,57,59], but the results regarding the measurement of other fractions are quite rare, fragmentary, and, sometimes, contradictory. Duff [36] observed that free fluoride added to milk tends to bind Ca^2+^ ions and/or unspecified organic components of milk. In particular, 72 h after the milk addition of known concentrations of fluoride ions, a fall to less than 10% of the initial free fluoride concentration is observed. Wieczorek et al. [64] investigated the tendency of α-lactalbumin, α-, β-, and κ-casein to combine with fluoride in the pH range from 6.6 to 3.9. They found that only α-lactalbumin can bind fluoride at pH = 3.9. Thus, fluoride preferentially binds the Ca^2+^ ions, that were released in solution by the proteins as a result of the pH decrease observed along the milk storage. On the contrary, Kahama et al. [46] found that almost all fluorine in cow’s milk was inorganic and the bound analyte was physically or chemically sequestered by milk proteins. Chlubek [42] reported that about 11% of fluorine in milk was bound to fat. Finally, Campus et al. [57] used an undescribed and unvalidated speciation protocol to differentiate organic and inorganic fractions of fluorine in research aimed at studying the effect of prepartum fluoride supplementation on breast milk.

From this evidence, a comprehensive and validated analytical protocol for differentiating and quantifying the most important forms of fluorine present in milk might be useful to ascertain the nature and the bioavailability of the element in this matrix. Unfortunately, this protocol is currently absent in the literature. Hence, pursuing the interest of this research group in the development and validation of original FISE methods applicable either in dental research [57,65,66,67,68] or in food analysis [69], the aim of this study is to develop for the first time (and validate, where possible) a comprehensive analytical protocol for the FISE determination of the fluorine contained in different fractions of bovine milk.

## 2. Results and Discussion

### 2.1. Classification of the Different Forms of Fluorine Present in Milk

Findings from the literature reveal that the same name has sometimes been used to indicate different forms of fluorine present in milk. To avoid any misunderstanding, it would be useful to clarify unambiguously the names of the most common forms of fluorine which have been measured in this study. In principle, inorganic fluorine is formed by free ionic fluoride (FIF) and inorganic bonded fluorine (IBF). FIF is the inorganic fraction that is directly measured in a solution (or suspension), consisting of equal volumes of the sample and a total ionic strength adjustment buffer solution (TISAB), and it can be found in the aqueous fraction of the milk. On the other hand, IBF is the fluorine fraction complexed by metal ions, or reversibly “adsorbed” by colloids, suspensions, and emulsions present in milk. The sum of FIF and IBF forms the total inorganic fluorine (TIF). The latter is the amount of analyte measured after the conversion of the free or bound inorganic forms of fluorine into HF, its diffusion in a siloxane medium, and its quantitative recovery in an alkaline solution. Non-diffusible, organic fluorine fractions in milk are mainly related to interactions of the analyte with proteins and lipids present in colloidal and emulsion particles, respectively. Proteins-bonded fluorine (PBF) is constituted by caseins-bonded fluorine (CBF) and whey protein-bonded fluorine (WBF), respectively. Furthermore, the sum of PBF and lipids-bonded fluorine (LBF) constitutes the total organic fluorine (TOF). Finally, the total fluorine (TF) is the amount of fluorine measured after a complete decomposition of the matrix (i.e., by microwave digestion or ashing).

### 2.2. Analytical Methods

The protocol of analytical methods proposed in this study consists of six methods, summarized in Table 1.

#### 2.2.1. Diffusible (Inorganic) Fluorine

##### Method M1—Determination of Free Ionic Fluoride (FIF)

Despite its apparent simplicity, only a few papers have accurately described FISE potentiometric methods dedicated to measuring FIF in milk. For example, Duff [36] determined ionic fluoride in bovine milk by simply diluting the sample in potassium nitrate and sodium citrate solutions. Instead, Koparal et al. [55] measured the same analyte in breast milk by mixing the sample with equal volumes of a TISAB III solution (i.e., a solution of ammonium chloride, ammonium acetate and *trans*-1,2-diaminociclohexane-N,N,N’,N’-tetraacetic acid monohydrate (CDTA) in water).

They showed that, to minimize bias, potentiometric determination of fluoride needs careful control of pH and ionic strength, as well as the elimination of any interfering species.

Therefore, pH must be buffered at 5.25, achieving in this way the best compromise in minimizing both HF and OH^−^ ion interferences. Similarly, high amounts of an inert electrolyte buffer the ionic strength of the final solution. Finally, small quantities of strong complexing agents are required to avoid the formation of stable complexes between the fluoride and free metal ions, such as Al(III), Fe(II), and Mg(II), whose concentrations in milk usually range between 10 μg dm^−3^ to 100 mg dm^−3^. Among the possible strong complexing agents, CDTA is one of the most effective in removing the interferences and it allows to form stable complexes at pH below neutrality (e.g., 5.25).

For this reason, the method used by Koparal et al. has been adopted in this protocol and fully validated.

Therefore, once the solutions reached the temperature of 20.0 ± 0.5 °C, the FISE potentiometric measurement was performed after mixing 10 cm^3^ of milk and 10 cm^3^ of a TISAB III solution in a 50 cm^3^ polyethylene beaker. After immersing both the FISE and the Ag/AgCl reference electrodes, the suspension was slowly stirred for at least 20 min until the equilibrium potential was reached.

##### Method M2-Total Inorganic Fluorine (TIF)

Since the methods proposed by Taves [37] and Liu [43] were insufficiently validated, the procedure used in this work for measuring TIF was an optimization of the one assessed by Kimarua et al. [44] and widely used by Kahama et al. [45,46]. First, 1 cm^3^ of milk was placed in a Petri dish with a hole in the lid. A polystyrene weighing boat containing 0.050 cm^3^ of 0.5 mol dm^−3^ NaOH (trap solution) was placed inside the dish. The plate was sealed around with a strip of Parafilm^®^, and 2 cm^3^ of a 4.0 mol dm^−3^ HClO_4_ solution in water saturated with hexamethyldisiloxane (HMDS) were added to the sample through the hole that was then closed with Parafilm^®^. After 18 h at 25 °C the trap solution was dissolved in 0.050 cm^3^ of 0.5 mol dm^−3^ HCl aqueous solution, and ultrapure water were added up to 2 cm^3^. Lastly, the potentiometric measurement was made according to the M1 method described above.

##### Method M3-Total Fluorine (TF)

The determination of the TF requires the conversion to fluoride of all the fluorine forms complexed or bounded with inorganic or organic compounds. To accomplish this, complete decomposition of the organic matrix is required. This is typically obtained through open incineration, fusion, or combustion procedures in an oxygen flask, oxygen bomb, or oxyhydrogen flame [16]. The determination of TF in different matrices has been thoroughly reviewed by Campbell [70]. In particular, TF in milk has been measured using incineration of the sample, followed by HDMS diffusion processes [42,46] and pyrohydrolysis (or hydrolysis with perchloric acid) [16]. Unfortunately, these methods are rather complex, time-consuming, and often not validated. Therefore, a microwave assisted milk acid digestion process is proposed in this protocol. First, 0.5 cm^3^ of the sample were placed in a perfluoroalcoxy ethylene (PFA) vessel and treated with 2 cm^3^ of 69% (*w*/*v*) HNO_3_. The microwave program included two steps, the first at 300 W for 30 s and the next at 600 W for 60 s. After digestion, the vessel was cooled to 4 °C overnight. Afterward, the cold solution was quickly treated with 2 cm^3^ of 50% (*w*/*v*) NaOH. The neutralized solution (typical pH of 5.5 ± 0.5) was diluted to 10 cm^3^ with ultrapure water and 1:1 mixed with a TISAB III solution for potentiometric analysis performed according to method M1.

To carefully estimate the amount of fluoride released from the inner walls of the PFA vessels after treatment with strong oxidizing agents [71], two blanks (0.5 cm^3^ of ultrapure water added with 2 cm^3^ of 69% HNO_3_) were subjected to the whole analytical procedure in the same vessel, both before and after the sample digestion. Then, the mean amount of fluoride released from each vessel was systematically subtracted from the fluoride concentration measured when the same vessel was used for milk digestion. The average concentration of fluoride for all blanks measured was 220 ± 80 μg dm^−3^ (n = 120).

#### 2.2.2. Non-Diffusible (Organic) Fluorine

As reported by Taves [37] and Singer [72], the concentration of TF is usually two times higher than TIF. As a result, other forms of fluorine, strongly interacting with the organic phase of milk and thus not available for diffusion, were postulated to explain the difference between the two concentrations.

After the early studies by Venkateswarlu [73], other researchers have studied the interactions between fluorine and proteins [46,63] or lipids [42]. Since caseins and whey proteins showed different interactions with fluorine [73], one of the aims of this study was the differentiation of fluorine bound to these proteins. Preliminary tests performed using a literature method [55] confirmed its known drawbacks of poor reproducibility and specificity [74,75]. Therefore, two original methods, based on the fractionate precipitation of caseins and whey proteins, have been developed.

First, caseins were precipitated to the isoelectric point [76], and fluorine previously bonded to them was released in solution as fluoride ion. From this solution, a salting out procedure [77] allowed the precipitation of the whey proteins and the consequential release in the solution of the fluorine previously bonded to them. Hence, the methods allowed to measure: (i) the sum of FIF and the organic fluorine associated with caseins (CBF), after their precipitation, and (ii) the sum of FIF and the fluorine fraction released to the total amount of milk proteins (PBF), after their precipitation. Finally, the amount of fluorine associated with lipids was measured by applying the method M3 on aliquots of pure lipid extracts obtained from milk according to the Rose-Gottlieb method [78].

##### Method M4—Free Inorganic Fluoride and Caseins-Bonded Fluorine (FIF + CBF)

The principle of the method is based on the precipitation of caseins to their isoelectric point. When this happens, calcium and fluoride ions bonded to caseins are released into solution [37]. Then, 10 cm^3^ of milk was added with a 0.5 mol dm^−3^ HCl aqueous solution until pH = 4.6 and transferred into a centrifuge tube. After 20 min centrifugation, the supernatant was filtered (solution A). An aliquot of solution A was added to the same volume of TISAB III solution for the potentiometric analysis, performed according to method M1.

##### Method M5—Free Inorganic Fluoride and Proteins-Bonded Fluorine (FIF + PBF)

After precipitation of caseins, the whey proteins were also precipitated from the solution by a salting out procedure [77]. To 5 cm^3^ of solution A, 4 cm^3^ of a saturated aqueous solution (26.8% (*w*/*v*)) of (NH_4_)_2_SO_4_ was added dropwise and under continuous stirring. The suspension was left stirring for 10 min. After 1 h centrifugation, the supernatant was filtered, and an aliquot of this solution was mixed 1:1 with TISAB III solution for the potentiometric analysis, performed according to method M1.

##### Method M6-Lipids-Bonded Fluorine (LBF)

The method is based on a preliminary extraction of milk lipids according to Rose-Gottlieb method [78] which are then processed with the method M3. The organic extract of lipids from milk was concentrated in a small volume (ca. 5 cm^3^) and quantitatively transferred in a PFA vessel. The vessel was gently heated up to 80 °C to allow the evaporation of the organic solvents. The residue was processed according to method M3.

#### 2.2.3. Quantification

Since some exploratory measurements on real samples revealed that the concentrations of the different forms of fluoride were below the lower limit of the linearity range of the M1-M6 methods (i.e., below the concentration of 100 μg dm^−3^), quantification has always been performed by means of internal calibration. Therefore, for each sample/blank analyzed, three known additions of a fluoride standard solution have been performed. Each addition was between 50% and 150% of the assumed amount of the analyte. The Gran’s-like linearization procedure provided the concentration of the analyte [79]. Compared with external calibration, the internal calibration method is more time-consuming, as each individual sample or blank requires a calibration curve [80], and it has a higher uncertainty due to the larger width of its confidence interval [81]. Despite these disadvantages, it allows for a substantially bias-free measurement [82]. To ensure the homogeneity of the results obtained in the proposed protocol, this quantification approach was used for all methods (i.e., M1–M6). All data were blank corrected.

The procedures aimed to ensure quality assurance and quality control of the methods have been described in detail in the Supplementary material (part S1).

### 2.3. Validation

Validation of the methods described in this protocol has been accomplished by means of the calculation of the limit of detection (LoD), limit of quantification (LoQ), and the measurement of both precision and trueness.

#### 2.3.1. LoD and LoQ

**Figure 1:**
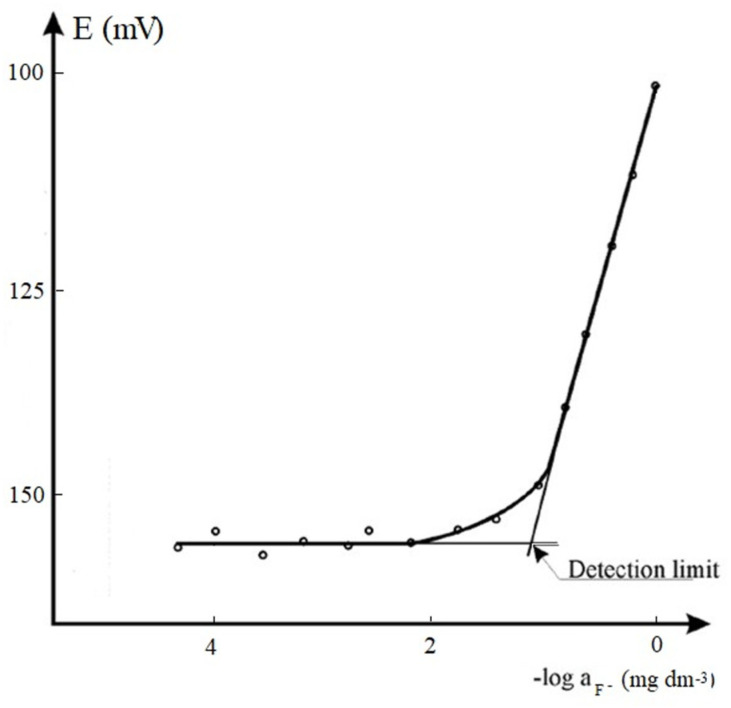
Calibration plot of standard fluoride solutions in the range between 5·10^−5^ mg dm^−3^ and 1 mg dm^−3^.

LoD of the potentiometric determination of free fluorides was determined using both the procedures described by the Laboratory Certification Program of the Wisconsin Department of Natural Resources (LCP-WDNR) [83] and by the International Union of Pure and Applied Chemistry (IUPAC) [84]. According to LCP-WDNR, the esteem of the standard deviation **s** of ten replicated measures on a standard fluoride solution at a concentration in the range between the expected LoD and 5LoD (in this case, 5·10^−2^ mg dm^−3^) was evaluated. Hence, LoD and LoQ are defined as LoD = s × t, and LoQ = 10 × s, respectively, where t is the tabulated Student’s t value for 9 degrees of freedom and p = 0.99. Based on the IUPAC method, LoD has been accomplished by measuring the potential of fifteen standard solutions at concentration levels between 5·10^−5^ (i.e., a concentration much below the expected LoD) and 1 mg dm^−3^. The LoD was calculated by plotting the two linear segments and corresponds to the abscissa of the intersection point. Figure 1 shows an example of the IUPAC approach for determining the LoD for a potentiometric method.

Despite the marked differences between the approaches used in these two methods, the LoDs reported in Table 2 are similar, and both are low enough to allow quantification of the analyte in each of the methods considered. Since the amount of fluorine measured in methods M1–M6 is always obtained by means of a potentiometric determination, the LoDs and LoQs here reported are the same for all methods proposed.

#### 2.3.2. Precision

Precision was evaluated for all methods in terms of intermediate precision, whereas the evaluation of the repeatability has been performed only for method M1. Intermediate precision, measured in terms of percent coefficient of variation (CV), has been evaluated analyzing the same milk sample in five analytical sessions performed within a week. Repeatability, expressed always as CV, and evaluated by means of eight consecutive determinations of FIF performed on the same milk sample, was 3.6%. Table 2 reports the intermediate precision data, which ranged between 7% for both methods M2 and M5, and 14% for method M4. The acceptability of the precision data for a concentration interval of analyte between the tens of the μg dm^−3^ (i.e., for FIF and LBF) and the hundreds of μg dm^−3^ (for the remaining methods) has been successfully verified in terms of the Horwitz’s theory [85].

#### 2.3.3. Trueness

Since certified reference materials (CRM) were not available and no reliable and analytical methods are reported in literature (except for methods M1 and M3), trueness has been determined using recovery tests for all the methods of the protocol.

After homogenization, three aliquots of a milk sample were enriched with increasing volumes of a fresh standard fluoride solution, while no analyte was added to the fourth aliquot of milk. Then, all aliquots were subjected to the full analytical procedure aimed at determining the specific form(s) of fluoride. The whole procedure was performed in triplicate. The recoveries reported in Table 2 ranged between 88 ± 9% (method M4) and 110 ± 10% (method M1). Quantitative recoveries (*t*-test, α = 0.05) were observed for all the methods. Hence, the release of fluorine-containing species from mineralization vessel material (i.e., PFA), which may be a potential source of bias in the methods M3 and M6, is well compensated by the subtraction of the blanks from the measured concentrations of both TF and LBF. However, to evaluate any other possible source of bias in the determination of the TF in milk, a real sample was analyzed with both method M3 and the incineration method proposed by Kahama et al. [46]. Additionally, in these cases, Table 2 reports quantitative recoveries for these methods.

In summary, the validation data of the proposed methods confirm that this protocol has good sensitivity and accuracy.

### 2.4. Application of the Protocol to Cow’s Milk

**Figure 2:**
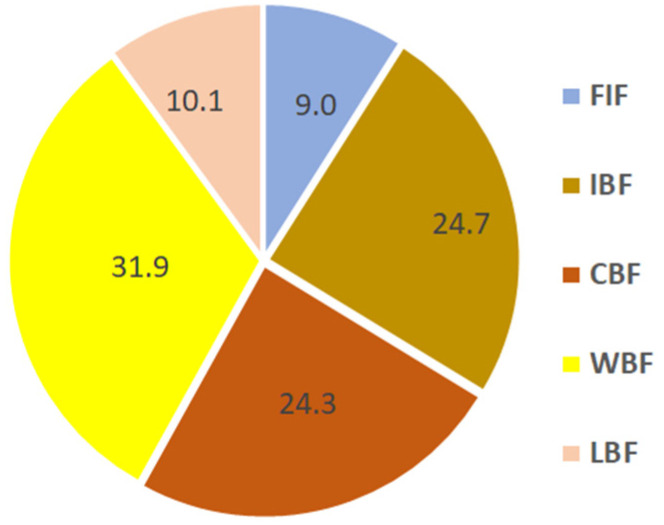
Average percent distribution of the different forms of fluorine in cow’s milk. FIF, free inorganic fluoride; IBF, inorganic bonded fluorine; CBF, caseins-bonded fluorine; WBF, whey-bonded fluorine; LBF, lipids-bonded fluorine.

The whole analytical protocol has been tested on five different samples of cow’s milk from markets in Sardinia. Table 3 reports the analytical data obtained both by the direct application of methods M1–M6 and indirectly by their combination, whereas Figure 2 reports the average percent distribution of the different forms of the analyte in cow’s milk.

The reported data clearly show that the FIF percentage is one-quarter of the TIF, being in turn minority (34%) regarding the TOF one (66%). Hence, the FIF paucity in milk (around 10% the amount of TF), already observed in the literature [36,86], has also been confirmed by these results. Furthermore, the very narrow interval of concentrations observed for FIF agrees with the range found for milk by Koparal et al. [55] and Liu et al. [43], but it is higher than those measured by Duff [36] and Bessho [87]. Additionally, TIF concentrations are comparable with those previously reported by Van Staden et al. [41], Kimarua et al. [44], and Kahama et al. [46] for milk produced in unpolluted sites.

Due to the paucity and fragmentary nature of previous studies on this topic, a comparison of the data obtained here for organic fluorine with those reported in literature is rather difficult. Normally, organic fluorine has been measured indirectly as the difference between total fluorine and total inorganic fluoride; therefore, these data are affected by high uncertainty and several possible sources of bias. To the best of our knowledge, only the contributions of Venkateswarlu [88] and Chublek [42] allow direct quantification of total organic fluorine in biological fluids (other than milk) and lipid-bound fluorine in milk, respectively. As would be expected, the indirect quantification of organic fluorine in milk is derived from literature studies that produced conflicting results. As a matter of fact, in some cases the amount of organic fluorine in milk was small [46] or even negligible [37], while in others it was the most abundant [57].

The percent of the TOF measured in this research is in good agreement with that reported in breast milk by Campus et al. [57] (i.e., 62%), however the fractionation method used was poorly described and validated and did not include the determination of LBF. Hence, the procedure of fractionated precipitation of caseins and globulins described in this protocol and used for the quantification of PBF, CBF, and WBF, has been described and validated for the first time. In addition, the preliminary data obtained in this study seem to envisage no significant statistical differences in the CBF/WBF ratio as a function of the origin of milk. Indeed, in cow’s milk the average ratio is 1.3, i.e., very close to the ratio of 1.27 measured by Campus et al. in the breast milk of mothers not involved in a fluoride supplementation study [57].

Furthermore, the results here obtained for the determination of LBF are in good agreement with those reported by Chlubek [42] (i.e., roughly 11% of the TF).

The TF amount measured by the method M3 ranged between 400 and 510 μg dm^−3^. These values are within the range measured by Obzek and Akman [49] as well as that reported by Liu et al. [43]. On the other hand, many previous contributions reported total amounts of fluoride in milk significantly lower [33,35,37,40,47] than those measured in this study. The fluorine concentration range in milk is generally wide, however, the rough validation of the analytical methods could have led to a general underestimation of the TF in milk. Acid-assisted mineralization methods and not optimized ashing methods [15] may cause losses of analyte fractions and lead to wrong measurements, where TF was really only TIF [43,45] or even FIF [51]. In this protocol, the trueness of the method for the TF determination has been carefully validated with a recovery test either using microwave digestion or milk ashing. These results were also confirmed by the substantial consistency for all samples among the sum of TIF and TOF with respect to TF. Hence, the percent ratio (TIF + TOF)/TF is between 101% and 112%.

## 3. Experimental

### 3.1. Samples

Semi-skimmed, UHT-treated, and long-shelf-life cow’s milk were purchased from local markets in North Sardinia, Italy. Sampling procedure has been described in detail in the Supplementary Materials (part S2).

### 3.2. Instrumentation and Labware

Potentiometric measurements were performed using a FISE (ISE Fluoride DX219, Mettler Toledo, Switzerland) connected to an Ag/AgCl reference electrode (mod. 373/SSG/6J, Amel s.r.l., Milan, Italy) and an ion analyzer (pH 1500 CyberScan, Eutech Instruments, Groningen, The Netherlands). The combined glass-electrode model LIQ-GLASS 238000/08 used for the pH measurements was from Hamilton, Bonaduz, Switzerland. A microwave digestion system Mega model MLS 1200 (Milestone, Sorisole, Italy), equipped with perfluoroalcoxy ethylene (PFA) medium-pressure digestion vessels, was used for the sample digestion. The thermostatic oven was purchased by Memmert, (Schwabach, Germany), whereas the centrifuge was by ALC 4217 MK II, ALC International s.r.l., (Cologno Monzese, MI, Italy). Fixed volume pipettes (0.500 and 1.000 cm^3^, Eppendorf, Milan, Italy; 5.000 and 10.000 cm^3^, Alpha, Rignano Flaminio, Italy), and 50 cm^3^ Falcon tubes in polypropylene were used for milk sampling. Polystyrene Petri dishes (55 mm) were used for microdiffusion measurements. Everywhere possible, glassware was replaced with plasticware.

### 3.3. Reagents

All reagents and solvents were of analytical-reagent grade (Fluka, Milan, Italy) except for NaF (99.99%, Sigma-Aldrich, Milan, Italy) and CH_3_COOH (100% extra pure, Riedel-de Haën, Milan, Italy). Type-I ultrapure water (resistivity >18 MΩ cm^−1^), used for all methods described in the protocol, was prepared with a Milli-Q^®^ IQ 7003 system (Millipore, Vimodrone, Italy). NaF was dried at 110 °C for two hours and cooled in a desiccator before preparation of 1000 mg dm^3^ F^−^ standard solution, which was used for preparing diluted solutions. TISAB III solution (ITW Reagents, Monza, Italy) consisted of 18 g of 1,2-diaminociclohexane N,N,N’,N’-tetraacetic acid 1-hydrate, 96.65 g of ammonium chloride, 163.4 g of ammonium acetate, 0.1 g of cresol red, all dissolved in 1 dm^3^ of ultrapure water. The pH of TISAB III is 5.25 ± 0.25.

## 4. Conclusions

For the first time, a complete protocol to quantify free ionic fluoride, total inorganic fluorine, total fluorine, caseins-bonded fluorine, proteins-bonded fluorine, lipids-bonded fluorine and, indirectly, inorganic bonded fluorine and whey-bonded fluorine has been developed, successfully validated in terms of LoD, LoQ, precision, and trueness, and tested on five different real samples of bovine milk. Among all fluorine fractions measured, free ionic fluoride is the least abundant, accounting for only 9% of the total amount of fluorine in milk. If other studies confirm this finding, there may be serious concerns about the efficacy of milk fluorination programs, since 66.3% of the total fluorine is bound to proteins or lipids, and for this reason is not available for human absorption. Beyond its novelty from a mere analytical viewpoint, this protocol might be a helpful tool for researchers engaged in studies on the bioavailability of fluoride in foods. In addition, the application of this protocol might be of interest to ascertain the authentic effectiveness of milk fluorination strategies, largely used for caries prophylaxis, and evaluating changes in the distribution of fluorine in proteins and lipids because of technological treatments. Furthermore, this protocol may be used—in whole or only for selected methods—in environmental monitoring procedures for fluorine-containing pollutants.

## Figures and Tables

**Table 1:** Summary of the protocol aimed to measure different forms of fluorine in cow’s milk.

Analytical Methods	Fluorine Fractions Measured	
M1	Free Ionic Fluoride (FIF)	FIF
M2	Total Inorganic Fluorine (TIF)	TIF
M3	Total Fluorine (TF)	TF
M4	FIF and Caseins-Bonded Fluorine (CBF)	FIF + CBF
M5	FIF and Proteins-Bonded Fluorine (PBF)	FIF + PBF
M6	Lipids-Bonded Fluorine (LBF)	LBF
**Indirectly measurable fractions of fluorine**		
M2-M1	Inorganic Bonded Fluorine (IBF)	IBF
M5-M4	Whey-Bonded Fluorine (WBF)	WBF

**Table 2:** Validation parameters for the methods M1–M6.

LoD and LoQ, μg dm^−3^	LoD ^a^		LoD ^b^		LoQ ^a^	
	1.3		6.6		4.0	
	M1	M2	M3	M4	M5	M6
Intermediate precision, CV%	10	7	10	14	7	12
Trueness, Recovery %	110 ± 10 ^c^	100 ± 10 ^c^	100 ± 10 ^c^ 100 ± 8 ^d^	88 ± 9 ^c^	90 ± 9 ^c^	100 ± 10 ^c^

^a^ according to ref. [83]; ^b^ according to ref. [84]; ^c^ measured using recovery test; ^d^ according to ref [46].

**Table 3:** Fluorine fractionation measured in five samples of cow’s milk from the market (n = 5). Concentration of each fraction is expressed as μg dm^−3^ ± standard deviation.

	Methods	Fractions	Samples					Average	Range ^b^
			1	2	3	4	5		
Direct	M1	FIF	42 ± 4	40 ± 3	43 ± 5	51 ± 4	47 ± 6	45 ± 10	40–51
	M2	TIF	180 ± 10	169 ± 4	136 ± 2	190 ± 20	160 ± 10	170 ± 20	136–182
	M3	TF	460 ± 80	450 ± 10	400 ± 60	510 ± 20	480 ± 30	500 ± 100	396–510
	M4	FIF + CBF	170 ± 10	150 ± 9	160 ± 20	180 ± 6	160 ± 10	160 ± 30	150–180
	M5	FIF + PBF	324 ± 6	335 ± 15	300 ± 20	319 ± 4	340 ± 10	320 ± 30	296–342
	M6	LBF	50 ± 10	50 ± 10	40 ± 10	60 ± 10	40 ± 10	50 ± 20	44–58
Indirect	M2−M1	IBF	140 ± 10	129 ± 5	93 ± 5	140 ± 20	110 ± 10	120 ± 30	93–140
	M4−M1	CBF	130 ± 10	110 ± 9	120 ± 20	129 ± 7	110 ± 10	120 ± 30	110–131
	M5−M1	PBF	282 ± 7	300 ± 20	250 ± 20	268 ± 6	300 ± 10	280 ± 30	268–295
	M5−M4	WBF	150 ± 10	180 ± 20	130 ± 30	139 ± 9	180 ± 20	160 ± 40	131–185
	M5−M1 + M6	TOF	330 ± 10	340 ± 20	290 ± 20	330 ± 10	340 ± 10	330 ± 30	302–343
	M5 + M6 + M2−M1	TIF + TOF	510 ± 20	510 ± 20	440 ± 20	510 ± 20	500 ± 20	500 ± 40	438–514
		(TIF + TOF) ^a^/TF (%)	110	112	110	101	105	108	

FIF, Free Inorganic Fluoride; TIF, Total Inorganic Fluorine; TF, Total Fluorine; CBF, Caseins-Bonded Fluorine; PBF, Proteins-Bonded Fluorine; WBF, Whey-Bonded Fluorine; TOF, Total Organic Fluorine; TF/(TIF + TOF) (%), percent difference among total fluorine directly measured with the method M3 and the sum of both TIF and TOF fractions. Average concentrations have been rounded according to the number of significant digits of the relevant standard deviation; ^a^ ratio calculated based on unrounded concentrations; ^b^ unrounded concentrations.

## Data Availability

Data is contained within the article or Supplementary Materials.

## Supplementary Materials

The following are available online at https://www.mdpi.com/article/10.3390/molecules28031349/s1. Part S1: Quality Assurance and Quality Control; Part S2: Sampling procedure of milk.

## Sample Availability

Samples are not more available from the authors.
