# Supplementary material for: An Analytical Protocol for the Differentiation and the Potentiometric Determination of Fluorine-Containing Fractions in Bovine Milk
Source: Molecules. 2023 Jan 31;28(3):1349. doi: 10.3390/molecules28031349 (PMC9921670; doi:10.3390/molecules28031349)
Supplement: Supplementary file 1 [file molecules-28-01349-s001.zip › molecules-2122966-supplementary.pdf]

molecules

MDPI

Article

An analytical protocol for the differentiation and the potentiometric determination of fluorine-containing fractions in bovine milk

Nadia Spano[1*], Sara Bortolu[2], Margherita Addis[3], Ilaria Langasco[1], Andrea Mara[1], Maria I. Pilo[1], Gavino Sanna[1*] and Pietro P. Urgeghe[4]

Supplementary material

Part S1. Quality Assurance and Quality Control

For quality assurance and control, the workflow of the analysis provided the analysis of three replicates for each sample and the analysis of a blank after a group of three samples. If the blank equilibrium potential differed by more than 5% of the average value of the same blanks, the FISE electrode underwent a complete cleaning cycle. It was performed by soaking for 30 minutes in a 0.1 mol dm^{-3} HCl aqueous solution containing 1% (w/v) of pepsin, followed by washing with ultrapure water and a final rubbing with an ethanol-wetted cloth. The cleaning cycle was always performed at the end of each session of measurements. No water washing was conducted between consecutive samples of milk belonging to the same group. In this way, dilution errors or possible modifications of the electrode surface were avoided, saving time to achieve the electrode equilibrium potential. When not used, FISE electrode was always stored dry in the air.

Part S2. Sampling procedure of milk

Packs sealed in unopened bags were held in the dark at room temperature until analysis. One hour before analysis, packaging was opened and a 50 cm^3 aliquot of milk was heated at 38°C and homogenized using an Ultraturrax for 15 minutes. Then, subaliquots of milk have been sampled for analyses with methods M1 to M6 by means of mechanical, fixed-volume pipettes of 0.500 cm^3, 1.000 cm^3, 5.000 cm^3, and 10.000 cm^3. After the opening of milk packaging, residual milk was transferred in several 50 cm^3 Falcon tubes and stored at -18° C.
